# An Experience

**Published:** 1869-01

**Authors:** C. M. Wright


### AN EXPERIENCE.

In the good old times of Methodism, and in the good old times of local Dental meetings, members used to get up in turns and give their Christian or their Dental experience (according to the meeting.) We would tell in one how the devil had, by a sharp trick, tempted us, and how we had, by having fortunately called to mind a verse from Scripture, resisted the flattering and seducing arts of the base foul fiend; and how he had, after a fling from us of some stinging hint, fled. We could describe, with tears of joy, how the old fellow had turned his spade-like tail and made rapid tracks with the heels of his hoofs toward us. In our Dental meetings, when then spirit of fraternal love warmed our blood, we would each arise and tell all about our methods or successes, our beliefs and hopes, and we would sometimes laughingly acknowledge a failure or two. This is certainly a good practice, and all who are not too well posted to be able to learn any thing new, enjoy these experience meetings and profit by them. But I'm not going to give a history of experience meetings, but will, with your permission, get up in these pages and give an experience with Morgan's Plastic Gold. I was a believer in the theory that has been expressed by many whose opinions are worth consideration, that crystal or plastic gold ought to be the best material for first-class contour fillings, and others, too, for that matter. I got some of Morgan's gold to try, it being highly recommended by names I had learned to regard. My first case was for filling two superior right bicuspids, approximal cavities. The teeth were badly decayed, and, when prepared for filling, left a very wide space between the cutting surfaces of the teeth. My gold was cut in lumps and blocks of various sizes, the plastic gold instruments were ready at hand, and a remarkably intelligent and muscular boy, with mallet raised, was standing by. All was ready and I went to work. Gradually the gold began to grow—the mallet taps rang like the blacksmith's hammer in the Anvil Chorus—a little time passed and behold, in place of a great hole and two absent halves of the two bicuspids, two beautiful, highly polished, richly colored gold halves seemed to have grown out of and upon the remnants of those crowns. "Ah! how beautiful," I exclaimed. I could not let the patient go till I had stood with clasped hands and glowing eyes before that gaping man, and admired the symmetry and perfectness, the beautiful and artistic results of my handiwork.

When the patient had been dismissed I cried "Eureka!" I had dreamed many a night, and oft, of just such fillings. I had made them in my slumbers, but never before in my waking hours. I had had an ideal plug floating before my mind's vision for months and years, but never had my eyes been permitted to feast in reality on such. My ideal was transplanted from the land of the imagination to the mouth of that plain man of the world, and *my* hand, with Morgan's gold, had done the thing. Oh! what joy! what happiness was mine. Do other men in other professions ever feel the exultant and triumphant, the jubalistic and tumultuous blaze of joy that occasionally falls to the Dentist's lot. Wherever I went I spoke in terms of praise of the plastic gold. My foil, I melted into plate and solder, in my laboratory, and I became an enthusiast. I was a Morgan's Plastic Gold man all over. I felt then, for the first time, as a brother Dentist once expressed it, "that it required genius to be an operator;" therefore, I am an operator, or a genius, I forget which. I continued the art of making artificial dentures as the trade of the "mere mechanic." Those were happy days for me, my brothers. Every case I had was only another opportunity for me to express my genius, by placing a beautiful gold substitute for every fraction of lost tooth, bone and enamel, and I accomplished the splendid results with plastic gold. I had read of the enthusiasms of men of genius, and I blessed Disraeli for having recorded and collected an account of them, and for his having told me of the absorption of all the faculties, and the concentration of them on one object, of the eagerness, the spirit, the spending of days and nights on a favorite subject or object, either in philosophy, science, poetry or art, of the minds of genius, and I felt and knew that I was a plugging genius. Future generations would acknowledge the greatness of my name, and bright pictures of fame encircled me. I was in danger of being called, like Raphael, "an enthusiastic madman," but little I recked if they'd let me plug on with the gold that Morgan had left us. In the hight of my enthusiasm, when, I'm afraid, I had silent contempt for all ye, my brothers, who were not using Morgan's gold, and, after about two or three months of happiness, my first patient called. I rushed toward him, seized his hands and begged to look at my first work as a genius. I dated the dawn of my genius from the time of filling those bicuspids. The coarse man did not seem to appreciate my cordiality, but rather doggedly opened his mouth, and, though I trembled with eager delight, when my eyes turned toward those bicuspids, my heart stopped beating. In agony I cried, "Where, oh where, are the beautiful monuments of my glory?" "Why, the infernal things crushed off in about two weeks, and the teeth have been giving me thunder ever since," was the reply of the unfeeling—*man*, must I say? Alas, it was true. A little spongy gold remained in each tooth, but decay had played around those little oozy balls, and the teeth were all but ruined. I was struck with mortification and astonishment, but as genius must suffer and be patient in secret, I excused myself for a moment, went into my library, tore out the music from a libretto of Faust, commencing "Gloria!" which I had been humming ever since I had been using Morgan's gold, bathed my throbbing temples, and returned to my patient cool and collected in manner. In a business like way I filled those teeth with amalgam, and suspended all opinions on every subject connected with Dentistry. In an hour and for a month, other patients called with fillings out, and fillings crushed and broken off, and defective, and pieces of gold out, &c. I had never been so busy. My business was immense. All work to be done over. Finally, my professional calls became so numerous, my practice so extensive, that my nervous system succumbed to the pressure, and I was compelled to retire from the active duties of the profession till health should again smile upon me.

Have others had such an experience? I hope not. Mine must be a bad and singular case. If it is, it lies with you who have been successful in the use of Morgan's Plastic Gold, to charge the fault to the operator. I only give my experience. I've also concluded that pride must have a fall, and to be struck with a genius for filling teeth is any thing but a blessing. C. M. WRIGHT.

